# Why Temporary Pacing in Acute Inferior Myocardial Infarction or No‐Reflow Becomes a Trigger—Not a Remedy—For Ventricular Fibrillation: Undersensing, “R‐On‐T”, Catheter Trauma and the Ischaemic Substrate

**DOI:** 10.1111/anec.70149

**Published:** 2026-01-09

**Authors:** Zhong‐Qun Zhan, Hai‐Jun Xu

**Affiliations:** ^1^ Department of Cardiology Shenzhen Guangming District People's Hospital Shenzhen P. R. China

**Keywords:** acute inferior myocardial infarction, no‐reflow phenomenon, temporary pacing, ventricular fibrillation

## Abstract

Temporary pacing during acute inferior MI or no‐reflow can trigger ventricular fibrillation rather than prevent it. Four mechanisms are highlighted: (1) acute ischemia lowers VF threshold and creates repolarization heterogeneity; (2) fragmented electrograms cause undersensing and asynchronous spikes; (3) bradycardia‐related long RR cycles position spikes on the T‐wave (“R‐on‐T”); and (4) catheter micro‐displacement induces mechanical extrasystoles. We propose a bedside decision framework—three questions before pacing—and a prevention bundle focused on urgent ischemia reversal, continuous electrogram surveillance, and early electrode removal. Bradycardia in this setting is often transient, but the electrophysiological vulnerability is not. Treating ischemia first and avoiding unnecessary pacing are paramount to prevent iatrogenic arrhythmia.

## Graphical abstract

Temporary pacing during acute inferior MI can paradoxically trigger VF via four mechanisms; the authors advocate reversing ischaemia before inserting a wire.

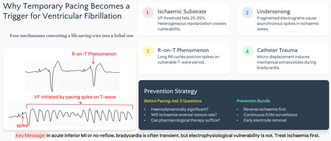

We read with interest the case report by Wu et al. (Wu et al. 2024) describing how acute right‐coronary no‐reflow after percutaneous coronary intervention produced transient pacemaker undersensing that initiated ventricular fibrillation (VF). Their example epitomizes a reproducible sequence that is still under‐appreciated at the bedside. We wish to highlight four intertwined mechanisms that convert a life‐saving wire into a lethal one in the very setting where it is most commonly used—acute inferior/right‐ventricular ischaemia or slow–/no‐reflow.

## 1 Ischaemia Creates the “Vulnerable Myocardium”

Within minutes of RCA occlusion or contrast‐induced no‐reflow, the ventricular fibrillation threshold in humans falls by 25%–35% (Lehmann et al. 1983).

In isolated canine sub‐endocardium, action‐potential amplitude decreases by ~50% within 3–5 min of ischaemia (Downar et al. 1977).

No‐reflow adds micro‐vascular obstruction and heterogeneous repolarisation—ideal milieu for re‐entry (Kaur et al. 2022).

## 2 Ischaemia Simultaneously Blinds the Pacemaker

Fragmented, low‐amplitude local electrograms—common when the electrode lies within or adjacent to an ischaemic zone—have been documented to cause intermittent undersensing, forcing the generator to default to asynchronous spikes (Wu et al. 2024; McLeod and Jokhi 2004).

## 3 Asynchronous Spikes Land on the T Wave (“R‐On‐T”)

Because inferior MI or no‐reflow often coexists with sinus bradycardia and prolonged RR intervals, the next pacing stimulus—whether from a fixed‐rate asynchronous mode or a poorly synchronized demand mode—may fall on the descending limb of the preceding T‐wave, that is, the ventricular vulnerable period. This “long‐cycle–short‐cycle” sequence, first described by Langendorf et al. in 1955 for spontaneous ectopic beats (Langendorf et al. 1955), was later documented in the setting of temporary ventricular pacing during acute MI (McLeod and Jokhi 2004).

## 4 Mechanical Catheter Trauma

Micro‐displacement of the floating catheter may produce mechanically induced extrasystoles; in the setting of sinus bradycardia or long RR intervals, these ectopic beats can fall on the descending limb of the preceding T‐wave (R‐on‐T), readily initiating ventricular fibrillation in the ischemic substrate (Lehmann et al. 1983; McLeod and Jokhi 2004).

## Before Insertion, Ask Three Questions

- Is the bradycardia haemodynamically significant?
- Will reversal of ischaemia (PCI, vasodilators, antithrombotics) restore adequate rate?
- Can pharmacological rate acceleration—such as atropine 0.5 mg IV, dopamine 5–10 μg k g^−1^ min^−1^, or aminophylline 150–300 mg IV over 15–30 min—suffice in haemodynamically stable patients with sinus bradycardia due to inferior MI or no‐reflow, absent high‐grade AV block? (Ibanez et al. 2018; Pasnoori and Leesar 2004; Kusumoto et al. 2021).

If the answer to any of these is “yes,” defer pacing, continue ischaemia‐directed therapy and reassess every 30–60 min. Temporary pacing is not a harmless bridge; it becomes unsafe when the myocardium is ischaemic (Lenarczyk et al. 2024).

## Prevention: Make the Myocardium Less Vulnerable and the Pacemaker Smarter

- Reverse ischaemia first—intracoronary adenosine, nitrates, aspiration thrombectomy, IABP if needed (Kaur et al. 2022).
- Continuous electrogram surveillance—No evidence‐based R‐wave threshold exists for the acute phase; monitor the pacing channel in real time and repeat sensing tests every 2–4 h or immediately after any arrhythmia. If undersensing is seen twice in a row, reposition the catheter.
- Display the pacing channel superimposed on the surface ECG for immediate visual recognition of spikes on the T wave.
- Pharmacological shield—Maintain serum K^+^ ≥ 4.0 mmol L^−1^ and Mg^2+^ ≥ 0.9 mmol L^−1^; in patients with recurrent VF or electrical storm, give magnesium sulfate 2 g IV over 5 min (EHRA 2024 consensus, Class IIa) (Lenarczyk et al. 2024).
- If VF occurs—defibrillate; immediately turn the pacemaker OFF; re‐evaluate sensing or reposition the catheter.
- Early exit strategy—once native rate > 50 min^−1^ and QRS morphology normalizes, taper the backup rate or remove the wire.

## Conclusion

In acute inferior MI or no‐reflow, bradycardia is often transient, but the electrophysiological vulnerability left by ischaemia is not. Temporary pacing under these circumstances is uniquely pro‐arrhythmic because the same process that slows the heart also lowers the VF threshold, sabotages pacemaker sensing, and provides a mechanical trigger. The remedy is to treat ischaemia first, verify robust sensing, avoid long escape intervals, and remove the wire as soon as perfusion is restored. The case by Wu et al. (Wu et al. 2024) is therefore a timely reminder: the most dangerous pacemaker is the one that paces without sensing—and without first trying to restore the heart's own rhythm.

## Author Contributions

**Zhong‐Qun Zhan:** conceived the commentary, drafted the manuscript, critically revised it for intellectual content, and approved the final version. Guarantor of the work. **Hai‐Jun Xu:** performed comprehensive literature review, analyzed data from published studies, contributed to manuscript drafting, created the graphical abstract concept, and approved the final version. Both authors agree to be accountable for all aspects of the work and ensure that questions related to the accuracy or integrity of any part of the work are appropriately investigated and resolved.

## Funding

This work was supported by the Shenzhen Guangming District Program for Introducing High‐Level Medical Teams (szgmtd2025004).

## Conflicts of Interest

The authors declare no conflicts of interest.

## Data Availability

The data that support the findings of this study are openly available in https://doi.org/10.1111/anec.70149.
